# Chinese Herbal Medicine for Diabetic Peripheral Neuropathy: An Updated Meta-Analysis of 10 High-Quality Randomized Controlled Studies

**DOI:** 10.1371/journal.pone.0076113

**Published:** 2013-10-16

**Authors:** Chi-zi Hao, Fan Wu, Lin Lu, Juan Wang, Yi Guo, Ai-ju Liu, Wei-jing Liao, Guo-qing Zheng

**Affiliations:** 1 Department of Rehabilitation, Zhongnan Hospital of Wuhan University, Wuhan, China; 2 Center of Neurology, The Second Affiliated Hospital of Wenzhou Medical College, Wenzhou, China; 3 Department of Epidemiology, School of Public Health, Wuhan University, Wuhan, China; 4 State Key Lab of Virology, Wuhan University, Wuhan, China; University of Lancaster, United Kingdom

## Abstract

**Background:**

Diabetic peripheral neuropathy (DPN) is very common in people with diabetes. Chinese herbal medicine (CHM) therapy has been developed for DPN empirically over the years. The aim of this systematic review and meta-analysis was to assess the efficacy and safety of CHMs for patients suffering from DPN.

**Methods:**

We performed a meta-analysis of randomized-controlled clinical trials (RCTs) evaluating the efficacy and safety of CHM on DPN. Six databases were searched up to November 2012. The primary outcome measures were the absolute values or changing of motor or sensory nerve conduction velocity (NCV), and the secondary outcome measurements were clinical symptoms improvements and adverse events. The methodological quality was assessed by Jadad scale and the twelve criteria recommended by the Cochrane Back Review Group.

**Results:**

One hundred and sixty-three studies claimed RCTs. Ten studies with 653 individuals were further identified based on the Jadad score ≥3. These 10 studies were all of high methodological quality with a low risk of bias. Meta-analysis showed the effects of NCV favoring CHMs when compared with western conventional medicines (WCM) (P<0.05 or P<0.01). There is a significant difference in the total efficacy rate between the two groups (P<0.001). Adverse effects were reported in all of the ten included studies, and well tolerated in all patients with DPN.

**Conclusion:**

Despite of the apparently positive findings and low risk of bias, it is premature to conclude the efficacy of CHMs for the treatment of DPN because of the high clinical heterogeneity and small sample sizes of the included studies. However, CHM therapy was safe for DPN. Further standardized preparation, large sample-size and rigorously designed RCTs are required.

## Introduction

Diabetic peripheral neuropathy (DPN) is one of the most common comorbidities of diabetes. DPN is a complex and progressive disorder, characterized by symmetrical distal degeneration of peripheral nerves, leading to symptoms of pain and sensory loss. As the disease progresses, symptoms can improve, predisposing the patients to diabetic ulceration and non-traumatic amputation [1].

The prevalence of DPN varies considerably depending on the diagnostic techniques used and patients selection. Annalisa [2] reported that 14.1% of people with diabetes had DPN in UK and 23.1% in Italy respectively. In the US, approximately one-third of people with diabetes aged 40 or older were diagnosed as DPN [3]. A recent study reported that 11% were diagnosed with DPN in young children, with short diabetes duration, and good diabetes control [4].

The pathophysiology of DPN is thought to be related to multiple factors, including glucose levels, metabolic and vascular factor, lifestyle, environmental factors, and inheritability. Although the exact pathogenesis is uncertain, persistent hyperglycemia is considered as a fundamental risk factor in the development of DPN [1], [5]–[6], and several studies have shown that strict glycemic control can reduce the occurrence and progression of DPN [7]–[9]. Currently, pharmacologic agents used in the treatment of diabetic neuropathy are used empirically to control the painful symptoms, including low-dose tricyclic antidepressants, anticonvulsants such as gabapentin, phenytoin, lamotrigine, opioids and tramadol, topical analgesic (topical capsaicin), and nonsteroidal anti-inflammatory drugs. Studies also demonstrated benefits of vitamin B12 on symptomatic improvement of patients with DPN [10]–[12]. Aerobic physical activity may be effective at improving peripheral nerve function and glycemic control of DPN patients, preventing the onset or modifying the natural history of DPN [13]–[15]. However, it remains largely underutilized because patient adherence is an issue.

Traditional Chinese medicine (TCM) including Chinese herbal medicine (CHM), acupuncture and other non-medication therapies has been used widely in China for thousands of years. CHMs therapy for DPN have been developed empirically over the years, and is now still widely used in China and elsewhere. The evidence from clinical studies suggested that CHMs could reduce the symptoms, improve nerve conduction velocity of patients with DPN [16]. Pharmacological studies demonstrated that CHMs could reduce oxidative stress and free radicals, and inhibit the apoptosis [17]–[18]; regulate the polyol pathway and related metabolic disorder, reduce the sorbitol content in cells [19]; activate protein kinase C [20]–[21]; inhibit the formation of advanced glycation endoproducts [22]; increase neurotrophy factors level such as BDNF, NGF and insulin-like growth factor-1 [21], [23]–[24]; improve haemodynamics, and decrease the levels of endothelin and ehromboxane [25]; decrease the production of inflammatory cytokines, and reduce the inflammatory reaction [26]. Recently, Sun et al. [27] reported that CHMs could exert the analgesic effect by reversing both the increased transient sodium currents and the reduced total potassium currents of painful diabetic neuropathy experimental rat model.

Owing to the significant health risk of DPN and the limitations of currently available conventional therapies, there have been a number of controlled studies over the past decade to evaluate the efficacy and safety of CHMs for DPN. Three systematic reviews addressing the efficacy of CHMs for DPN have also been published recently [28]–[30], and concluded that the total efficacy rate and NCV in CHMs group were better than that in control group. However, their conclusions are not scientifically sound because most of the primary trials included were of low methodological quality and the small number of trials were included in the meta-analysis. Moreover, many new data have been published. Therefore, it is worthwhile to undertake an update systematic review and meta-analysis to assess the efficacy and safety of CHMs for patients suffering from DPN.

## Methods

### Eligibility Criteria

#### Types of Studies

We selected randomized controlled clinical trials (RCTs) that compared any CHM with non-CHM interventions for DPN patients, and included high-quality RCTs with Jadad score_3 or above in efficacy and safety analysis. Quasi-RCTs were not considered such as using the admission sequence for treatment allocation.

#### Types of Participants

Patients of any gender, age, or race/ ethnicity with diabetic peripheral neuropathy were included. The definition of diabetic neuropathy used in the studies had to accord with the following diagnostic criteria: (1) diabetes mellitus was diagnosed according to the internationally recognized criteria, such as the World Health Organization criteria [31] or the American diabetes association criteria [32]; (2) the patient had a predominantly distal symmetrical sensorimotor polyneuropathy of the limbs, including subjective complaints of pain, tingling, numbness, weakness, and reduced functioning of the peripheral nerves demonstrated by a nerve conduction test; (3) other causes of sensorimotor polyneuropathy were excluded.

#### Types of Interventions

The patients of the control group were given no intervention, placebo or conventional medicines. The patients at the treatment groups were given CHM interventions. We also included trials of Chinese herbal medicine plus conventional medicine versus conventional medicine alone. Studies comparing one with another form of CHM were excluded. The clinical trials were included regardless of length of treatment period and dosage of treatment. The CHM interventions were included regardless of single herbs, a compound of several herbs or a Chinese proprietary medicine. The mode of delivery was restricted to orally.

#### Types of Outcome Measures

The primary outcome measures of interest were the absolute values or changing of motor or sensory nerve conduction velocity after treatment. The secondary outcome measurements were clinical symptoms improvements such as the total efficacy rate, and adverse events reported in the study. Clinical efficacy is defined as the ability of CHM to prevent or reverse clinical symptoms related to DPN. The TCM syndrome score criteria of DPN were adopted based on Guideline for Clinical Trials of New Patent Chinese Medicines [33], including limb pain, numbness, sensory disturbances, dry mouth and polydipsia, fatigue and weakness, soreness and weakness of waist and knees, feverishness in palms and soles, tongue and fur, and pulse manifestation (Table 1). The effective rate was conducted in accordance with the TCM syndrome score criteria [33], which classified clinical therapeutic effects into four categories as cure (TCM clinical symptoms and signs disappeared or almost disappeared, the TCM syndrome scores were decreased up to 91–100%), significant improvement (TCM clinical symptoms and signs significantly improved, the TCM syndrome scores were decreased at 71–90%), improvement (TCM clinical symptoms and signs improved, the TCM syndrome scores were decreased at 31–70%), no improvement (The TCM clinical symptoms and signs were not improved or aggravated, the TCM syndrome scores were decreased less than 30%). Moreover, it was dichotomized as effective (including the categories of cure, significant improvement, and improvement) and ineffective (including the category of no improvement). Other assessment criteria of clinical therapeutic effect made by other authors with comparable definitions were also considered.

**Table 1:** The TCM syndrome score criteria of diabetic peripheral neuropathy.

Items	TCM syndrome score criteria			
	Normal (0 point)	Mild (2 points)	Moderate (4 points)	severe (6 points)
Limb pain	asymptomatic	slight and occasional limb pain	persistent limb pain that can be endured, and does not affect sleep	persistent limb pain, unbearable, and severe sleep disturbance
Numbness	asymptomatic	slight and occasional numbness of toe/finger	sustained numbness of toe/finger, not obvious after distracting	sustained numbness below the elbow/knee
Sensory disturbances	asymptomatic	pain/temperature sensation subsided to the wrist	pain/temperature sensation subsided to knee/elbow	cold hands and feet to the wrist/Elbow or knee/ankle
Dry mouth and polydipsia	asymptomatic	dry mouth, slightly increased water intake	dry mouth, increased water intake half times more than past	dry mouth, increased water intake than past 1 times or more
Fatigue and weakness	asymptomatic	(mild limb weakness, climbed upstairs with heavy legs	limb weakness sometimes mild and sometimes severe, heavy legs when walking the ground	significant limb weakness, lower limb heaviness obviously when lifting the legs
Soreness and weakness of waist and knees	asymptomatic	occasional soreness and weakness of waist and knees	frequently soreness and weakness of waist and knees, can participate in activities of daily living	continuous soreness and weakness of waist and knees, liking to lay in bed
Feverishness in palms and soles	asymptomatic	occasional feverishness in palms and soles	someone who want to expose his/her own hands and feet outside bedding and clothing, frequently upset	feel comfortable only when hands and feet approached the cold object, vexation and restless
Tongue and fur	asymptomatic	deep purple tongue or ecchymosis on tongue or tortuous, bluish and purplish sublingual collateral vessel		
Pulse manifestation	gentle and regular	stringy pulse or deep and hesitant pulse or thready pulse		

### Information Sources and Search

We searched Cochrane library, PubMed, EMBASE, China National Knowledge Infrastructure, VIP Journals Database, and Wanfang database until November 2012. The search strategy combined two facets: the condition (DPN) and the intervention (Chinese herbal medicine). The search terms used were (herbal-medicine OR herbs OR Chinese herbal medicine OR Chinese medicinal herb) AND (diabetic peripheral neuropathy OR diabetic neuropathy OR DPN). Chinese Databases were also searched using the above search terms in Chinese. We hand-searched Chinese journals that may publish potentially eligible studies and conference proceedings relevant to this topic. The reference lists of all relevant articles were searched for further studies.

### Study Selection and Data Collection Process

All articles were screened by two independent reviewers (Hao C.Z., Wu F.), who extracted data from the articles according to a standardized data extraction form, including patients, methods, interventions and outcomes. The reasons for inclusion and exclusion of studies were recorded accordingly at all stages. For eligible studies, two review authors (Hao C.Z., Wu F.) extracted the data independently. Disagreements were resolved through consultation with a third party author (Guo Y. or Zheng G.Q.).

### Risk of Bias in Individual Studies

For each included study, two reviewers (Hao C.Z., Wu F.) independently completed the Jadad scale for assessing methodological quality. Trials scoring 1 or 2 points are considered low quality and 3–5 points as high quality [34]. And the risk of bias was further assessed using the twelve criteria recommended by the Cochrane Back Review Group [35], the items were scored with “yes (+)”, “no (−)”, or “unsure (?)”. Studies were categorized as having a “low risk of bias” when at least six of the 12 criteria were met. Disagreements were resolved by discussion between the two reviewers (Hao C.Z., Wu F.), with the opinion of a third party author (Guo Y. or Zheng G.Q.) if necessary.

### Description of the CHMs

The selection criteria of high-frequency herbs in Treatment of DPN were those with cumulative frequencies over 50%.

### Summary Measures and Synthesis of Results

We synthesized the results in a meta-analysis. A fixed-effects model or random-effect model was used across the trials, and risk ratios with their 95% confidence intervals (CI) were calculated for dichotomous data. If continuous data were available, weighted mean difference or standardized mean difference was to be calculated using RevMan 5.1 software provided by the Cochrane Collaboration. Heterogeneity between trial results was tested using a standard chi-square test and we also calculated the I^2^ statistic. The two-tailed P values less than 0.05 were considered statistically significant. Where possible, we assessed potential publication bias using a funnel plot.

## Results

### Study Selection

**Figure 1:**
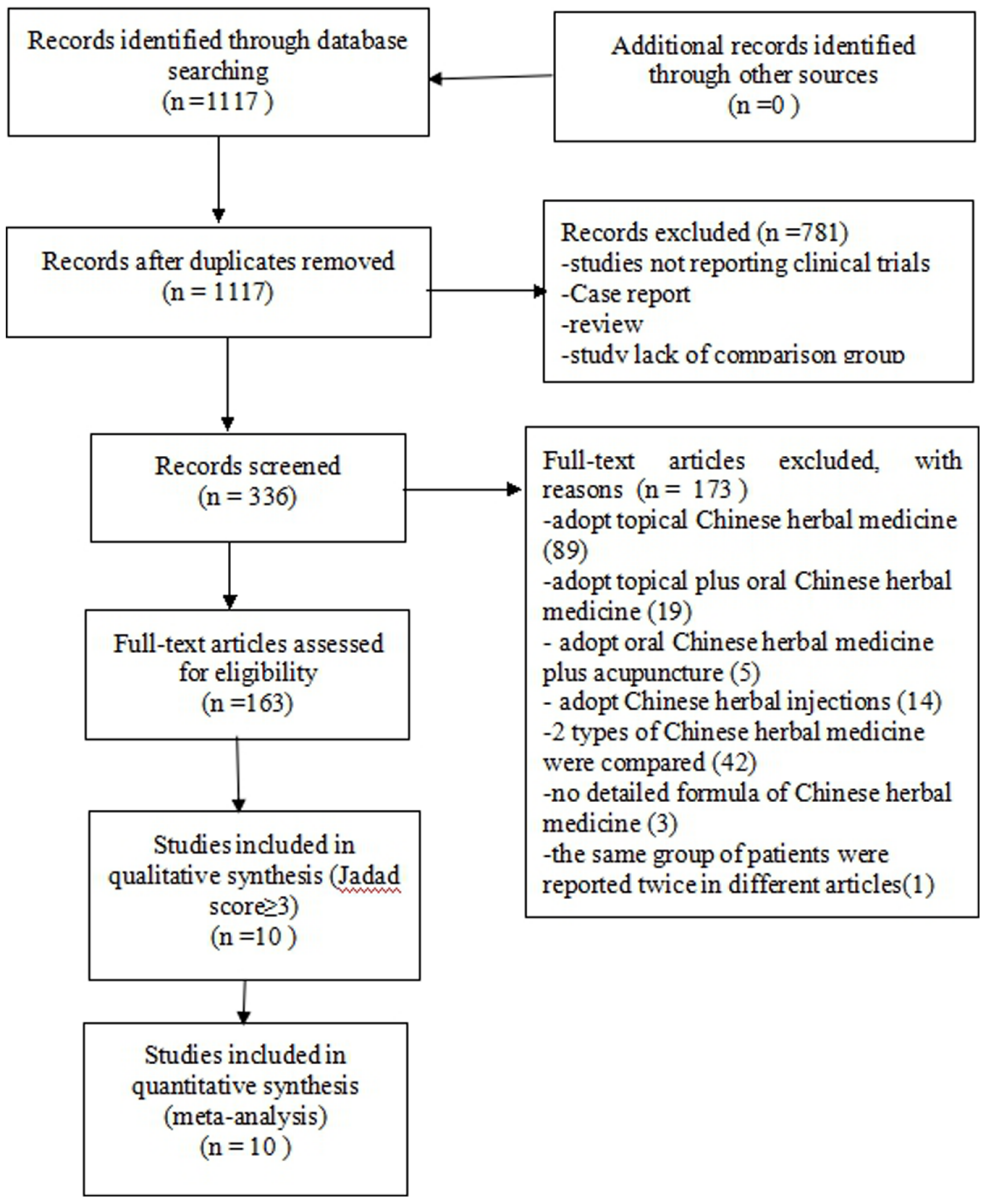
PRISMA 2009 Flow Diagram.

We identified 1117 potentially relevant articles, and 781 articles were excluded because they were not reporting clinical trials, review, case report, or lacking comparison group. Of the remaining 336 articles, 173 were excluded because 89 adopted topical CHM in the treatment group, 19 adopted topical plus oral CHM, 5 adopted oral CHM plus acupuncture, 14 adopted Chinese herbal injections, 42 compared one type of CHM to another, 3 had no information about the formula of CHM, and 1 reported the same group of patients with another included article. Finally, 163 studies were left and assessed by the Jadad score. Ten articles scoring ≥3, involving a total of 653 participants met our inclusion criteria [36]–[45]. The screening process is summarized in a flow diagram (Figure 1).

### Study Characteristics

The 10 studies included were all conducted in China and published between 2004 and 2012. Eight studies were performed in a single center while the other two studies [43]–[44] were performed in multicenter. A total of 653 participants of Chinese ethnicity were included in the 10 studies, of whom 316 were male and 302 were female (the gender of the left 35 participants could not be obtained from the primary data) ranging from 25 to 71 years old. Among the 10 included studies, one was three-group design study, while the remaining 9 were two-group parallel design studies. The diagnostic criteria were based on the World Health Organization (WHO) criteria in 8 studies [37]–[43], [45], and American Diabetes Association (ADA) criteria in the other 8 studies respectively [36], [44].

Seven trials evaluated the effects of CHM compared to mecobalamin [36]–[38], [40]–[41], [44]–[45]. However, the specific compositions of these herbal formulae were different in each of the 7 trials. One trial compared the effects of CHM plus pancreatin tablets to pancreatin tablets alone [39], and 2 trials evaluated the effects of the same CHM capsule compared to inositol [42]–[43]. Hypoglycemic therapy was used as a co-intervention in all the 10 included trials, including oral hypoglycemic drugs, insulin treatment, and exercise. The duration of treatment lasted from 6 weeks to 12 weeks. Adverse effects were reported in all of the 10 trials. Detailed characteristics of included studies were listed in Table 2 and detailed compositions of CHM of included studies were listed in Table 3.

**Table 2:** Summary of the characteristics of the included trials and the assessment of methodology.

First author year	Subjects (T/C)	Age (T/ C)	Duration of diabetes	Duration of DPN	Intervention		Main Outcome measures	Course of treatment (d)	Adverse events	Follow up
					T	C				
Hu2012	30/30	59.17±9.92/5 9.77±6.26	8.71±6.32/ 9.01±5.49	4.52±3.06/ 4.36±3.44	CHM	mecobalamin	TER	12w	4/0	n.r.
Chen2011a	17/10	63.52±5.21/61.17±7.38	n.r.	5.63±5.35/5. 72±6.05/	CHM	mecobalamin	TER, NCV	12w	No	n.r.
Chen2011b	17/8	63.52±5.21/6 1.73±5.75	n.r.	5.63±5.35/3. 70±4.05	CHM	placebo	TER, NCV	12w	No	n.r.
Cao2011	30/30	60.06±4.73/5 9.10±4.82	7.94±3.13/ 7.71±3.32	2.54±1.60/2. 63±2.09	CHM	mecobalamin	TER, NCV	6w	No	n.r.
Li 2011	30/30	58.30±8.33/5 8.37±9.52	9.17±4.36/ 9.53±4.13	n.r.	CHM+	pancreatin tablets	TER, NCV	8w	No	n.r.
Zhou 2010	20/20	58.4±10.67/6 1.7±4.92	n.r.	5.85±2.71/4. 95±2.74	CHM	mecobalamin	TER, NCV	8w	No.	n.r.
Zhang 2008	38/36	61.19±8.59/6 1.67±9.22	10.77±4.78 /9.88±4.95	2.93±2.07/2. 61±2.22	CHM	mecobalamin	TER	8w	No.	n.r.
Liu 2004	24/24	56.33±7.73/5 5.04±6.61	7.35±3.91/. 48±3.73	2.63±2.08/2. 46±1.27	CHM	inositol	TER, NCV	8w	No	n.r.
Heng 2004	60/60	55.5±6.3/54.8±6.6	7.7±3.2/7.6±2.9	2.3±1.6/2.3±1.5	CHM	inositol	TER, NCV	8w	19/20	n.r.
Li 2005	55/53	48.3±14.9/45 ±20.27	12.1±7.3vs 11.5±7.78	4.7±0.6/4.9±0.6	CHM	mecobalamin	TER, NCV	12w	No.	n.r.
Liu 2005	24/24	51.31±6.47/5 1.84±7.01	12.52±3.98 /11.31±3.05	4.52±1.21/4. 46±1.12	CHM	mecobalamin	TER, NCV	8w	No	n.r.

Notes: T: Trial Group, C: Control Group, +: mean same as the control group treatment; TER: Total efficacy rate, NCV: Nerve conduction velocity; n.r.: not report; No: no adverse event was identified; Chen2011a: CHM compared with mecobalamin; Chen2011b: CHM compared with placebo.

**Table 3:** Herbal medicines in the included studies.

First author year	Name of Herbs	Formulation	Compositions	Usage
Hu2012	Guizhi Shaoyao Zhimu Tang\	decoction	cassia twig (Ramulus Cinnamomi), debark peony root (Radix Paeoniae Alba), ephedra (Herba Ephedrae), largehead atractylodes rhizome (Rhizoma Atractylodis Macrocephalae), common anemarrhena rhizome (Rhizoma Anemarrhenae), divaricate saposhnikovia root (Radix Saposhnikoviae), prepared common monkshood branched roo (Radix Aconiti Lateralis Preparata), fresh ginger (Rhizoma Zingiberis Recens), liquorice root (Radix Glycyrrhizae), suberect spatholobus stem (Caulis Spatholobi), danshen root (Radix Salviae Miltiorrhizae)	100ml, Tid,
Chen2011	Xiaoke Tongluo Capsule	capsule	milkvetch root (Radix Astragali seu Hedysari), red ginseng (Radix Ginseng Rubra), danshen root (Radix Salviae Miltiorrhizae), sanqi (Radix Notoginseng), unprocessed rehmannia root (Radix Rehmanniae Recens), peony root (Radix Paeoniae Rubra), leech (Hirudo)	4 capsules, Tid,
Cao2011	YiqiWenyanghuoxue Tang	decoction	milkvetch root (Radix Astragali seu Hedysari), cassia twig (Ramulus Cinnamomi), sichuan lovage rhizome (Rhizoma Ligustici Chuanxiong), earthworm (Lumbricus), suberect spatholobus stem (Caulis Spatholobi), peony root (Radix Paeoniae Rubra), debark peony root (Radix Paeoniae Alba), medicinal cyathula root (Radix Cyathulae), unprocessed rehmannia root (Radix Rehmanniae Recens), manchurian wildginger (Herba Asari)	100ml, Tid,
Li 2011	Buqi Huoxue Xiaobi Tang	decoction	milkvetch root (Radix Astragali seu Hedysari), Chinese angelica (Radix Angelicae Sinensis), peony root (Radix Paeoniae Rubra), sichuan lovage rhizome (Rhizoma Ligustici Chuanxiong), earthworm (Lumbricus)	100ml, Tid,,
Zhou 2010	Tongluo Tangtai Tang	decoction	milkvetch root (Radix Astragali seu Hedysari), common yam rhizome (Rhizoma Dioscoreae), figwort root (Radix Scrophulariae), dwarf lilyturf tuber (Radix Ophiopogonis), danshen root (Radix Salviae Miltiorrhizae), sichuan lovage rhizome (Rhizoma Ligustici Chuanxiong), suberect spatholobus stem (Caulis Spatholobi)	150ml, Tid
Zhang 2008	Tangluoning Capsule	capsule	milkvetch root (Radix Astragali seu Hedysari), unprocessed rehmannia root (Radix Rehmanniae Recens), cibot rhizome (Rhizoma Cibotii), twotoothed achyranthes root (Radix Achyranthis Bidentatae), danshen root (Radix Salviae Miltiorrhizae), sichuan lovage rhizome (Rhizoma Ligustici Chuanxiong)	100ml, Bid
Liu 2004	Tangluotong Capsule	capsule	leech (Hirudo), White Mustard Seed (semen brassicae), borneol (Borneolum Syntheticum), American ginseng (Radix Panacis Quinquefolii), Chinese angelica (Radix Angelicae Sinensis), yanhusuo (Rhizoma Corydalis), figwort root (Radix Scrophulariae), golden thread (Rhizoma Coptidis)	n.r.
Heng 2004	Tangluotong Capsule	capsule	leech (Hirudo), White Mustard Seed (semen brassicae), borneol (Borneolum Syntheticum), American ginseng (Radix Panacis Quinquefolii), Chinese angelica (Radix Angelicae Sinensis), yanhusuo (Rhizoma Corydalis), figwort root (Radix Scrophulariae), golden thread (Rhizoma Coptidis)	2 capsules, Tid
Li 2005	Tangpingluotong Yin	decoction	milkvetch root (Radix Astragali seu Hedysari), heterophylly falsestarwort root (Radix Pseudostellariae), kudzuvine root (Radix Puerariae), unprocessed rehmannia root (Radix Rehmanniae Recens), leech (Hirudo), peony root (Radix Paeoniae Rubra), sichuan lovage rhizome (Rhizoma Ligustici Chuanxiong), suberect spatholobus stem (Caulis Spatholobi), Chinese starjasmine stem (Caulis Trachelospermi), honeysuckle stem (Caulis Lonicerae), twotoothed achyranthes root (Radix Achyranthis Bidentatae), peach seed (Semen Persicae), frankincense (Olibanum), myrrh (Myrrha)	100ml, Tid,
Liu 2005	Modified Huangqi Guizhi Wuwu Tang	decoction	milkvetch root (Radix Astragali seu Hedysari), cassia twig (Ramulus Cinnamomi), peony root (Radix Paeoniae Rubra), earthworm (Lumbricus), suberect spatholobus stem (Caulis Spatholobi), common yam rhizome (Rhizoma Dioscoreae), liquorice root (Radix Glycyrrhizae)	100ml, Tid,

### Description of the CHMs

Thirty-nine herbs were included in the 10 studies with Jadad scored ≥3. The top 11 most frequently used herbs were ordinally milkvetch root (Radix Astragali seu Hedysari), suberect spatholobus stem (Caulis Spatholobi), peony root (Radix Paeoniae Rubra), sichuan lovage rhizome (Rhizoma Ligustici Chuanxiong), danshen root (Radix Salviae Miltiorrhizae), leech (Hirudo), unprocessed rehmannia root (Radix Rehmanniae Recens), cassia twig (Ramulus Cinnamomi), earthworm (Lumbricus), Chinese angelica (Radix Angelicae Sinensis), figwort root (Radix Scrophulariae), which were used more than 3 times (Table 4).

**Table 4:** Analysis of the top 11 frequency Chinese herb medicine in treatment of diabetic peripheral neuropathy.

Herb name English (Latin)	Frequency	The total frequency %	Cumulative percentiles %
ordinally milkvetch root (Radix Astragali seu Hedysari)	7	8.43	8.43
suberect spatholobus stem (Caulis Spatholobi)	5	6.03	14.46
peony root (Radix Paeoniae Rubra)	5	6.03	20.49
sichuan lovage rhizome (Rhizoma Ligustici Chuanxiong)	5	6.03	26.52
danshen root (Radix Salviae Miltiorrhizae)	4	4.82	31.34
leech (Hirudo)	4	4.82	36.16
unprocessed rehmannia root (Radix Rehmanniae Recens)	4	4.82	40.98
cassia twig (Ramulus Cinnamomi)	3	3.61	44.59
earthworm (Lumbricus)	3	3.61	48.20
Chinese angelica (Radix Angelicae Sinensis)	3	3.61	51.81
figwort root (Radix Scrophulariae)	3	3.61	55.42

### Risk of Bias within Studies

The methodological quality of each study was assessed using the Jadad score and all the included trials appeared to have a high quality with Jadad score varing from 3 to 5. And the risk of bias was further assessed using the twelve criteria recommended by the Cochrane Back Review Group. The number of criteria met varied from 6/12 to 10/12, which indicating that all of the included trials having a low risk of bias based on the Cochrane Risk of Bias tool. More details on the scores for each trial were present in Table 5.

**Table 5:** The methodological quality of the included trials.

First author year	12-item criteria													Jadad scale					
	A	B	C	D	E	F	G	H	I	J	K	L	T	a	b	c	d	e	T
Hu2012	+	+	-	-	?	+	+	?	+	+	+	+	8	1	1	0	0	1	3
Chen2011	?	?	+	+	?	-	+	?	+	+	+	+	7	1	0	1	1	1	4
Cao2011	+	?	+	?	?	-	-	?	+	+	+	+	6	1	1	1	0	0	3
Li 2011	+	?	-	-	?	+	+	?	+	+	+	+	7	1	1	0	0	1	3
Zhou 2010	+	?	-	-	?	-	+	?	+	+	+	+	6	1	1	0	0	1	3
Zhang 2008	+	?	-	-	?	-	+	?	+	+	+	+	6	1	1	0	0	1	3
Liu 2004	+	+	+	+	?	-	-	?	+	+	+	+	8	1	1	1	1	0	4
Heng 2004	+	+	+	+	?	+	+	?	+	+	+	+	10	1	1	1	1	1	5
Li 2005	+	?	-	-	?	-	+	?	+	+	+	+	6	1	1	0	0	1	3
Liu 2005	+	?	-	-	?	-	+	?	+	+	+	+	6	1	1	0	0	1	3

A to L, the 12-item criteria. A, adequate sequence generation; B, concealment of allocation; C, blinding (patient); D, blinding (investigator); E, blinding (assessor); F, incomplete outcome data addressed (ITT analysis); G, incomplete outcome data addressed (drop-outs); H, free of selective reporting; I, similarity at baseline; J, co-interventions constant; K, compliance acceptable; L, timing outcome assessments.

a to e, the Jadad scale. Points were awarded as follows: a, study was described as randomized, 1 point; b, appropriate randomization method, 1 point; c, study described as double-blinded, 1 point; d, appropriate double-blinded method, 1 point; e, description of withdrawals and dropouts, 1 point. The Jadad scale score ranges from 1 to 5; higher scores indicate better quality of the randomized controlled trial (RCT). T total.

### Effectiveness

**Figure 2:**
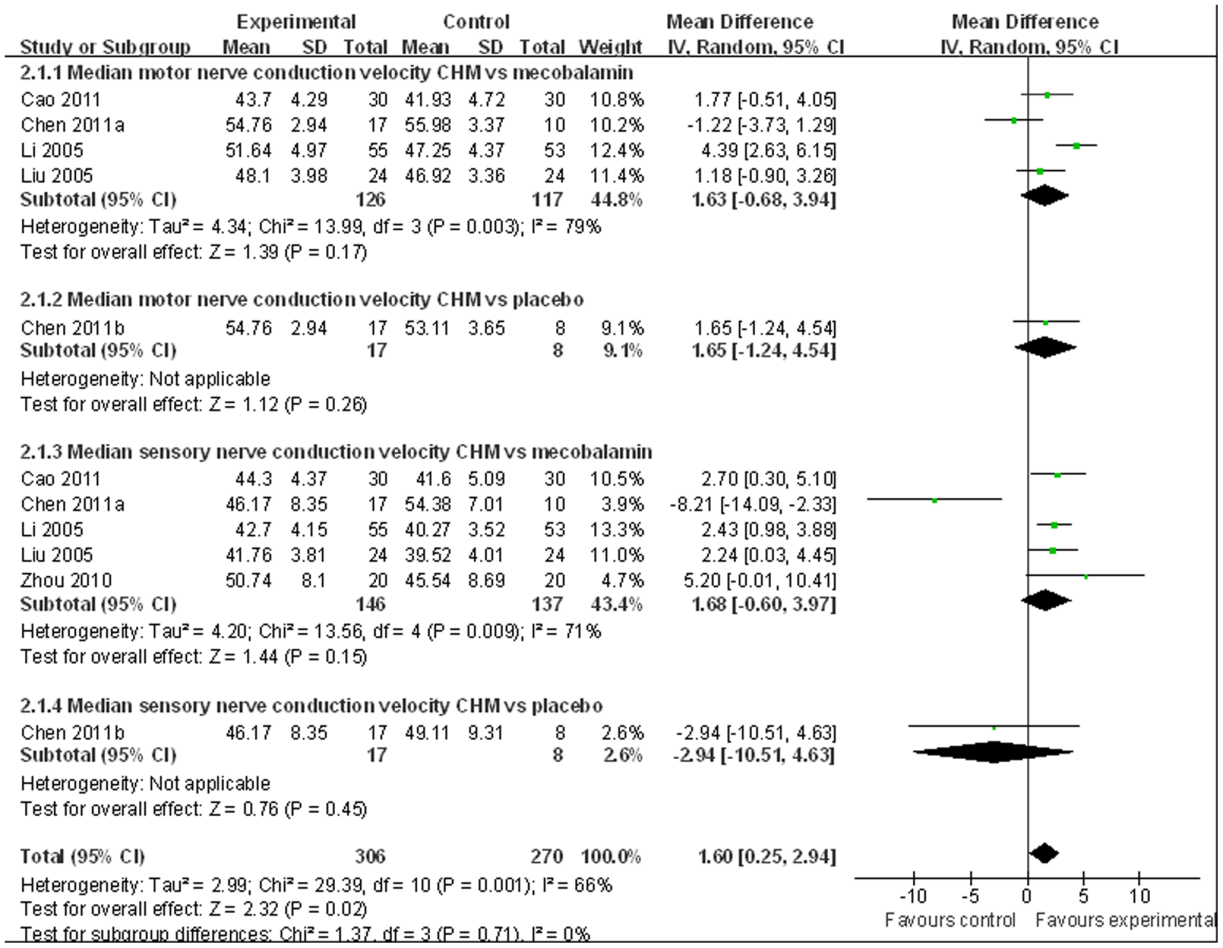
Forest plot of median nerve conduction velocity of CHM for diabetic peripheral neuropathy.

**Figure 3:**
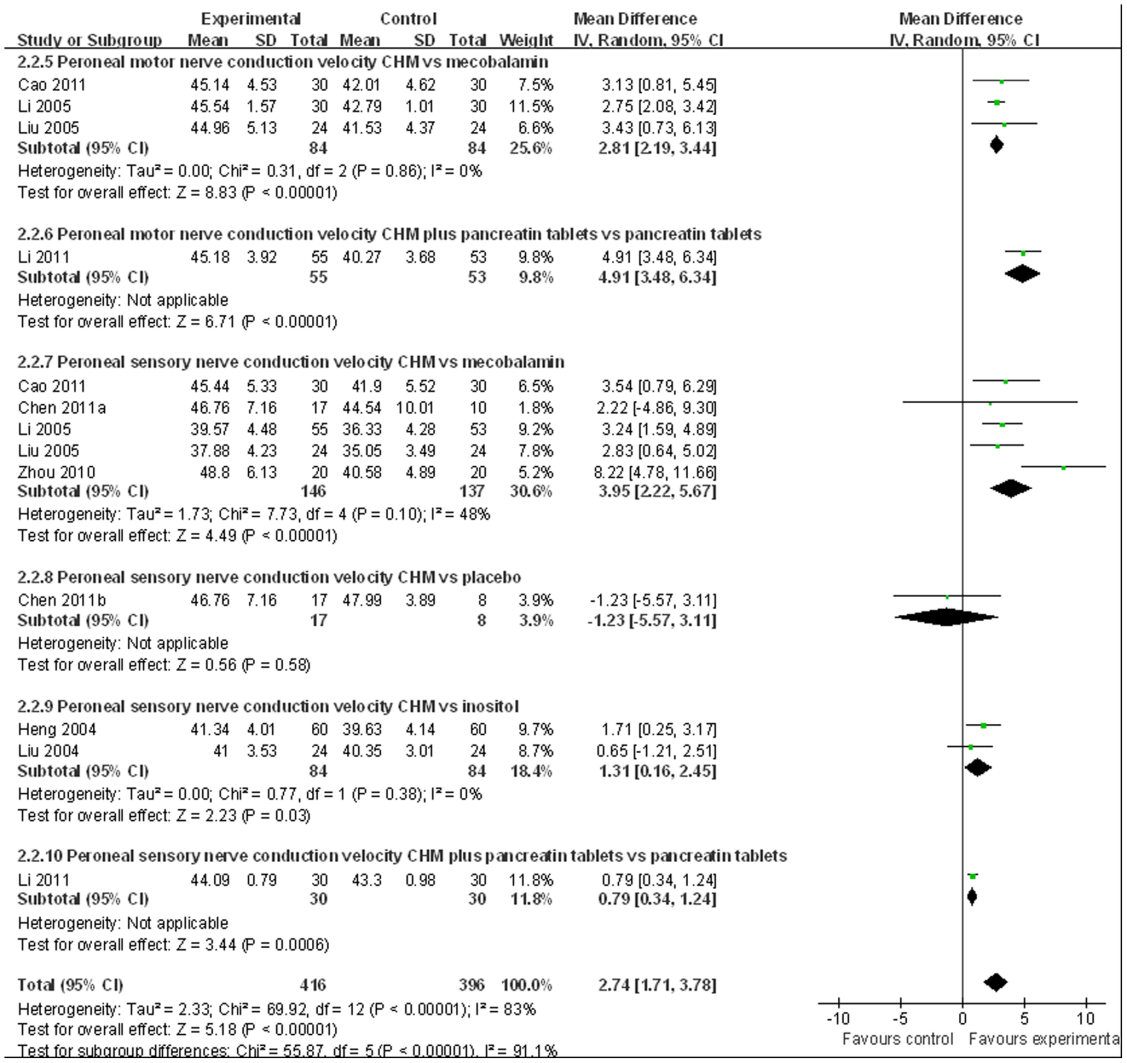
Forest plot of peroneal nerve conduction velocity of CHM for diabetic peripheral neuropathy.

**Figure 4:**
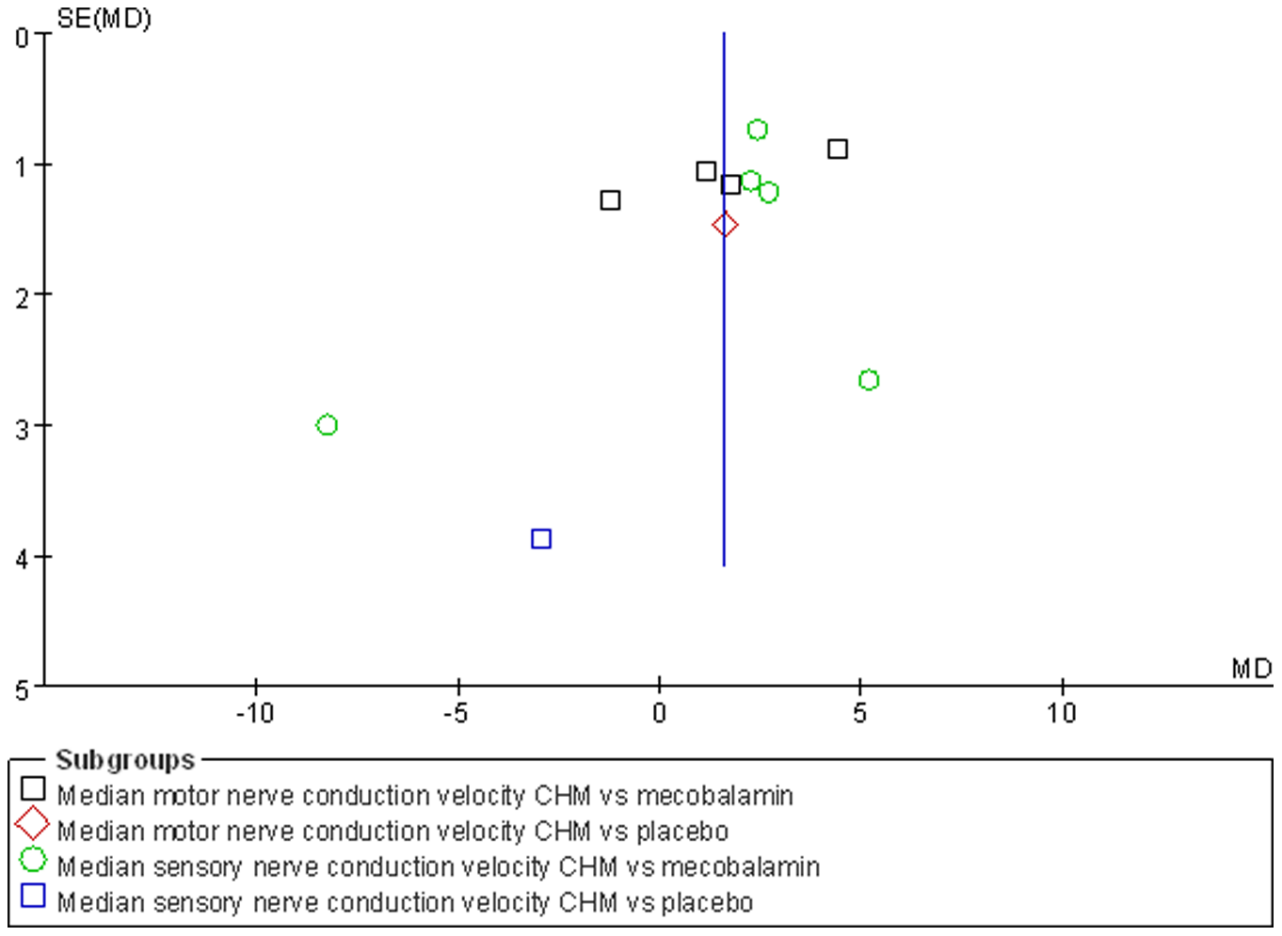
Funnel plot of median nerve conduction velocity of CHM for diabetic peripheral neuropathy.

**Figure 5.Funnel:**
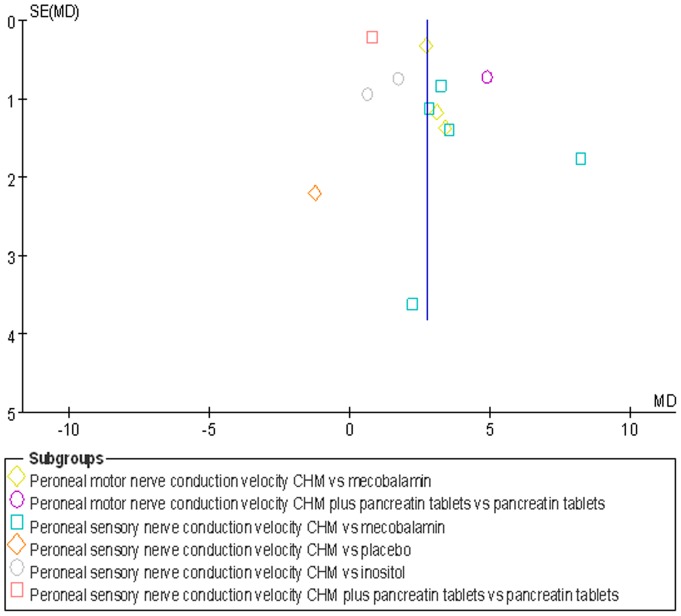
plot of peroneal nerve conduction velocity of CHM for diabetic peripheral neuropathy.

Nerve conduction velocity (NCV) was observed in 8 [37]–[40], [42]–[45] of the 10 included studies, including 7 CHM monotherapy studies [37]–[38], [40], [42]–[45] and 1 CHM adjuvant therapy study [39]. Four CHM monotherapy studies compared the effect on median motor nerve conduction velocity (MMNCV) with the mecobalamin control [37a, 38, 44–45] and 1 with the placebo control [37b], the combined effects showed that CHM had a significantly better effect on MMNCV than mecobalamin control (n = 243, mean difference (MD) 1.63, 95% CI: −0.68–3.94, P = 0.17, heterogeneity chi-square  = 13.99, P = 0.003, I^2^ = 79%) and placebo control (MD 1.65; 95% CI −1.24 to 4.54). The combined effects showed that CHM monotherapy [37a, 38, 40, 44–45] had a significantly better effect on median sensory nerve conduction velocity (MSNCV) when compared with mecobalamin (n = 283, MD 1.68, 95% CI: −0.60–3.97, P = 0.15, heterogeneity chi-square  = 13.56, P = 0.009, I^2^ = 71%) but not a favorable effect when compared with placebo [37b] (MD −2.94; 95% CI −10.51 to 4.63) (Figure 2). Three CHM monotherapy studies [38], [44]–[45] compared the effect on peroneal motor nerve conduction velocity (PMNCV) with the mecobalamin control group, the combined effects showed that CHM had a significantly better effect on PMNCV (n = 168, MD 2.81, 95% CI: 2.19–3.44, P<0.00001, heterogeneity chi-square  = 0.31, P = 0.86, I^2^ = 0%). One CHM adjuvant therapy study [39] compared the effect on PMNCV with the pancreatin tablets and the result showed that CHM had a significantly better effect on PMNCV (MD 4.91; 95% CI 3.48 to 6.34). 8 CHM monotherapy studies compared the effect on peroneal sensory nerve conduction velocity (PSNCV) with the mecobalamin [37a, 38, 40, 44–45] or placebo [37b] or inositol [42], [43] control group, the combined effects showed that CHM had a significantly better effect on PSNCV than mecobalamin control (n = 283, MD 3.95, 95% CI: 2.22–5.67, P<0.00001, heterogeneity chi-square  = 7.73, P = 0.10, I^2^ = 48%) and inositol control (n = 168, MD 1.31, 95% CI: 0.16–2.45, P = 0.03, heterogeneity chi-square  = 0.77, P = 0.38, I^2^ = 0%) except placebo control (MD −1.23; 95% CI −5.57 to 3.11). The CHM adjuvant therapy study [39] compared the effect on PSNCV with t pancreatin tablets control group showed that CHM had a significantly better effect on PSNCV (MD 0.79; 95% CI 0.34 to 1.24) (Figure 3). The publication bias funnel plot provided evidence of publication bias (Figure 4 and Figure 5).

**Figure 6:**
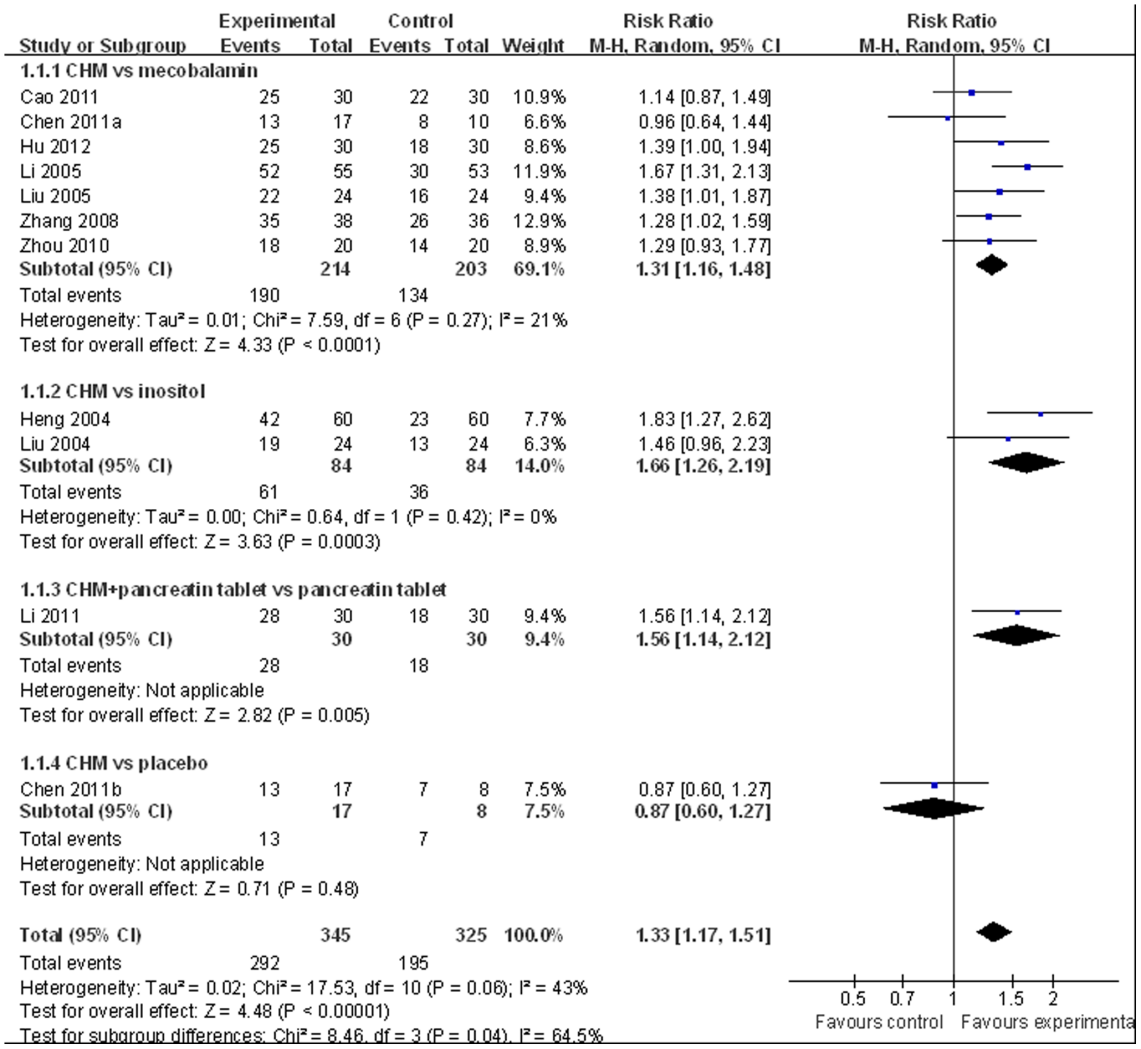
Forest plot of efficacy rate of CHM for diabetic peripheral neuropathy.

**Figure 7:**
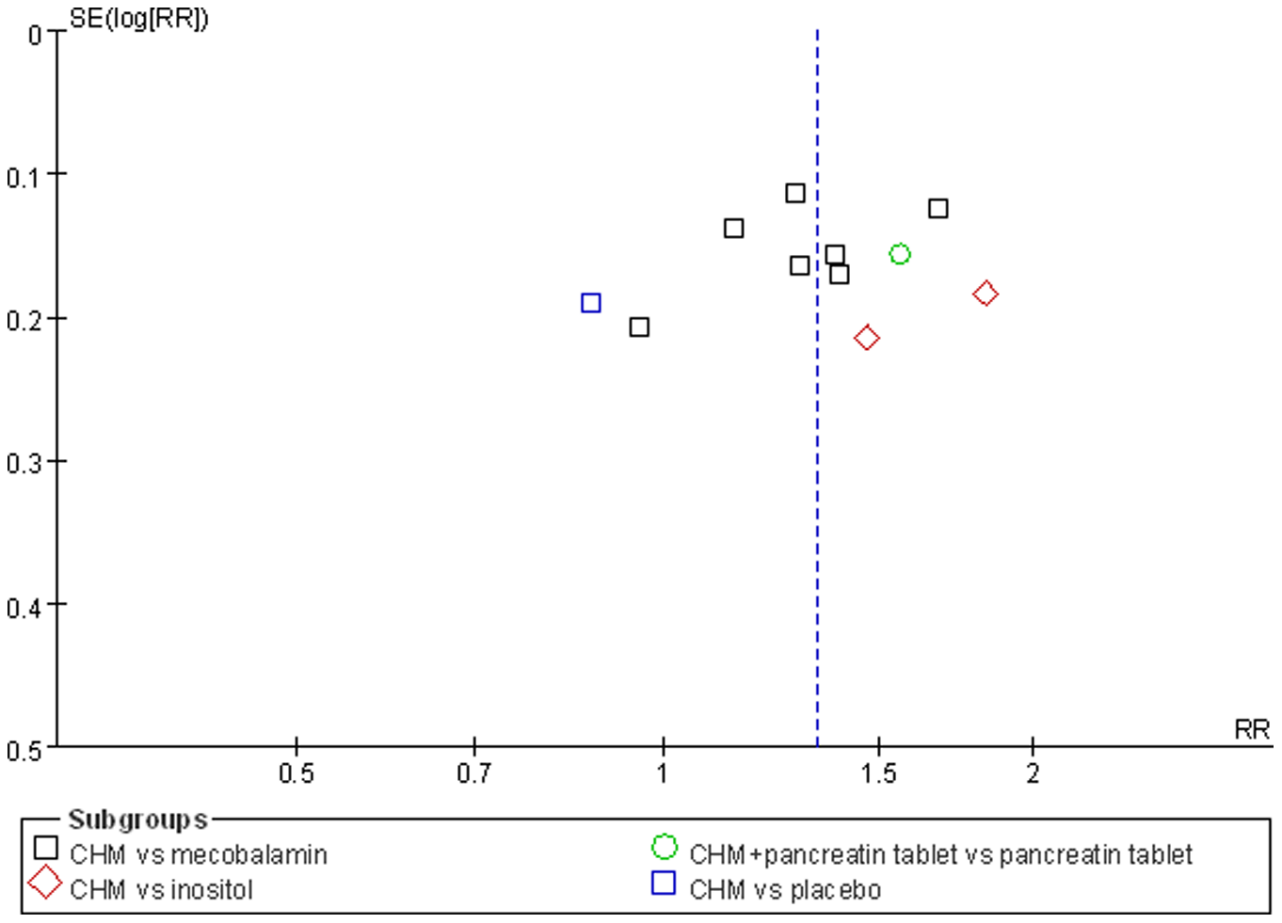
Funnel plot of efficacy rate of CHM for diabetic peripheral neuropathy.

Total efficacy rate were assessed in all the 10 included studies, including 9 CHMs monotherapy studies and 1 CHM adjuvant therapy study. The combined effects showed that CHM monotherapy had a significantly total efficacy rate when compared with mecobalamin [36]–[38], [40]–[41], [44]–[45] (n = 417, relative risk (RR): 1.31, 95% confidence interval (CI): 1.16–1.48, P<0.00001, heterogeneity chi-square  = 7.59, P = 0.27, I^2^ = 21%) and inositol [42], [43] control (n = 168, RR 1.66, 95% CI 1.26–2.19, P = 0.0003, heterogeneity chi-square  = 0.64, P = 0.42, I^2^ = 0%). However, CHM monotherapy did not show a favorable effect on total efficacy rate when compared with placebo [37] (RR 0.87; 95% CI 0.60 to 1.27). In CHM adjuvant therapy study, Li et al. [39] indicated that CHM adjuvant therapy had a significantly better effect on total efficacy rate than pancreatin tablets (RR 1.56; 95% CI 1.14 to 2.12) (Figure 6). The publication bias funnel plot provided evidence of publication bias (Figure 7).

Adverse effects were reported in all of the included studies, and no life threatening adverse effects were noted in all studies. Most of the trials (8/10) mentioned that no obvious adverse effects were found both in CHM group and control group. One study [36] reported that 4 cases suffered from upset and sweating in CHM group, and no obvious adverse effects were found in control group. Another study reported that side effects were 31.7% and 33.3% for CHM monotherapy and inositol control respectively [43], including mild abdominal pain, diarrhea, nausea, chest discomfort. The above results suggested that CHM monotherapy and adjuvant therapy were relatively safe for DPN.

## Discussion

### Summary of Evidence

This study is the update meta-analysis of English and Chinese literature to determine the efficacy and safety of CHM for DPN. One hundred and sixty-three studies claimed RCTs. Ten high quality studies with 653 individuals were identified based on the Jadad score ≥3. The main findings were that CHM monotherapy and adjuvant therapy could improve the clinical symptoms and NCV of DPN, and had fewer adverse effects in comparison with WCM controls. Despite of the apparently positive findings, it is premature to conclude the efficacy of CHMs for the treatment of DPN because of the high clinical heterogeneity of the included studies and small number of trials in the meta-analysis. Adverse effects were reported in all of the included studies, and CHM used generally appeared to be safe and well tolerated in patients with DPN in all studies. Thus, we can make it as a conclusion that CHMs therapy was safe for DPN.

### Limitations

There are a number of limitations to this review. Firstly, none of included studies had been registered. In September 2004, the members of the International Committee of Medical Journal Editors (ICMJE) published a statement requiring that all clinical trials must be registered in order to be considered for publication [46]. However, none of included studies in this review had been formally registered in WHO International Clinical Trials Registry Platform. Thus, protocols were not available to confirm free of selective reporting.

Secondly, although the methodological quality of the included RCTs was generally high according to the Jadad scale and the twelve criteria recommended by the Cochrane Back Review Group, there were still some methodological weaknesses in the primary studies. Most of the included studies (9/10) provided sufficient information on how the random allocation was generated, but only 3 trials described allocation concealment, which may produce selection bias. Four-tenth studies mentioned subjects blinding, and 4/10 mentioned investigator while no study described assessor blinding. Only 3/10 studies described intention-to-treat analyses, and no study reported follow-up data. Therefore, the results generated from these studies should be interpreted with caution.

Thirdly, among the 10 included studies, only one [37] used a formal placebo control. All of the left 9 studies included in this review used an ‘‘A or A + B versus B’’ design in which patients were randomized to receive a CHM monotherapy or an adjuvant therapy with CMH plus WCM versus WCM control treatment, without a rigorous control for placebo effect. Because of the lack of placebo controls, the interpretation of the positive findings of treatment with CHM should be made with caution.

Fourthly, clinical efficacy rate was used as the major outcome measures to show effectiveness in this review, which was measured through subjective qualitative scores such as “cure”, “significant improvement”, “improvement”, and “no improvement”. The 4 classifications for overall symptom improvement as an outcome measure were commonly used in Chinese trials but not internationally recognized, which may limit the validity and reliability of the outcome. Moreover, the time of the measurement was different among the trials, which leads to difficulties in interpreting the effects. Nerve conduction velocity was another weakness in the primary studies. All of the included studies adopted the treatment duration of 6–12 weeks. We should be cautiously interpret this outcome because it was more reliable if the changing of NCV was measured when the treatment duration lasted more than 12 weeks. At last, all the studies met the criteria coming from China was another weakness that potentially limited the generalizability of the findings.

Fifthly, the clinical heterogeneity compromised the validity of the included studies. There were large variations in the formulation, dosage, administration, and duration of treatment in the CHM of included studies. Moreover, several forms of CHM were tested in the included studies lacking detailed information about quality control for manufacturing methods and standards of CHM, which is crucial for the validity of the study results. Future studies should provide sufficient information about standardization in terms of formulation, quality control, purity, dosage, administration, and duration of treatment [47].

Sixthly, most of the included studies were of relatively small sample size and without formal sample size estimation. Trials with inadequate sample sizes often run the risk of overestimating intervention benefits [48]. The results were likely to be underpowered [49].

### Implications for practice

This is update systematic review of randomized, controlled trials to assess the efficacy and safety of CHM for DPN. Due to the high clinical heterogeneity of the included studies and the small sample sizes of trials included in this systematic review, the current evidence is insufficient to recommend the routine use of CHM for DPN. However, CHMs appeared to be well tolerated in all included studies. Thus, CHM therapy was safe for DPN. However, all trials were performed only on Chinese people. No trials testing the drug on other ethnic groups were found. Thus, the results may have limitations for generalizing to populations out of China.

### Implications for research

CHM is widely used in the treatment of DPN. Although the present evidence is insufficient for support efficacy of CHM, it is a promising candidate for further clinical trial of DPN. The most frequently used herbs such as milkvetch root, Chinese angelica, sichuan lovage rhizome, peony root, danshen root, earthworm, suberect spatholobus stem, safflower, cassia twig, unprocessed rehmannia root, peach seed, liquorice root, kudzuvine root, leech, debark peony root may contribute in composing a fundamental prescription for clinical DPN treatment. Since the concern in methodological quality, we recommend that the CONSORT 2010 statement [50], [51], which consists of a 25-item checklist to determine study quality and rigor, should be used as a guideline when further designing and reporting RCTs. In addition, sufficient information about formulation, quality of the preparations, purity, dosage, administration, and duration of treatment should be provided in future studies [47].

## Conclusion

In spite of the apparently positive findings based on the 10 high quality studies, there is insufficient evidence regarding the efficacy of CHM for the treatment of DPN because of the high clinical heterogeneity of the included studies and small sample sizes of the included trials. Adverse effects were reported in all of the included studies, and CHM was generally safe. Therefore, we can arrive at a conclusion that CHM therapy was safe for DPN. Further standardized preparation, large sample-size and rigorously designed RCTs are required.

## Supporting Information

Appendix S1 **Search strategies.**
(DOCX)

Appendix S2 **PRISMA checklist.**
(DOC)

## References

[1] Boulton AJ, Vinik AI, Arezzo JC, Bril V, Feldman EL, et al.. (2005) American Diabetes Association. Diabetic Neuropathies A statement by the American Diabetes Association. Diabetes Care. 28: 956–962. PubMed: 15793206. 10.2337/diacare.28.4.956 15793206

[2] Rubino A, Rousculp MD, Davis K, Wang J, Bastyr EJ, et al (2007) Diagnosis of diabetic peripheral neuropathy among patients with type 1 and type 2 diabetes in France, Italy, Spain, and the United Kingdom. Prim Care Diabetes 1: 129–134 10.1016/j.pcd.2007.07.006.PubMed:18632033 18632033

[3] Agrawal Y, Carey JP, Della Santina CC, Schubert MC, Minor LB (2010) Diabetes, vestibular dysfunction, and falls: analyses from the National Health and Nutrition Examination Survey. Otol Neurotol 31: 1445–1450 10.1097/MAO.0b013e3181f2f035.PubMed:20856157 20856157

[4] Moser JT, Langdon DR, Finkel RS, Ratcliffe SJ, Foley LR, et al (2013) The evaluation of peripheral neuropathy in youth with type 1 diabetes. Diabetes Res Clin Pract 100: e3–6 10.1016/j.diabres.2013.01.015.PubMed:23391743 23391743

[5] Perkins BA, Greene DA, Bril V (2001) Glycemic control is related to the morphological severity of diabetic peripheral sensorimotor polyneuropathy. Diabetes Care 24: 748–752. PubMed: 11315842. 10.2337/diacare.24.4.748 11315842

[6] Wooten K (2009) Clinical features and eletrodiagnosis of diabetic peripheral neuropathy in the dysvascular patients. Phys Med Rehabil Clin N Am 20: 657–676 10.1016/j.pmr.2009.06.011.PubMed:19781504 19781504

[7] Dahl-Jørgensen K, Brinchmann-Hansen O, Hanssen KF, Ganes T, Kierulf P, et al.. (1986) Effect of near normoglycaemia for two years on progression of early diabetic retinopathy, nephropathy, and neuropathy: The Oslo study. Br Med J (Clin Res Ed). 293: 1195–1199. PubMed: 3096429. 10.1136/bmj.293.6556.1195 PMC1341978 3096429

[8] Diabetes Control and Complications Trial Research Group (1993) The effect of intensive treatment of diabetes on the development and progression of long-term complications in insulin-dependent diabetes mellitus. N Engl J Med 329: 977−986. PubMed: 8366922. 10.1056/NEJM199309303291401 8366922

[9] UK Prospective Diabetes Study (UKPDS) Group (1998) Intensive blood-glucose control with sulphonylureas or insulin compared with conventional treatment and risk of complications in patients with type 2 diabetes (UKPDS 33). Lancet 352: 837−853. PubMed: 9742976. 9742976

[10] Sun Y, Lai MS, Lu CJ (2005) Effectiveness of vitamin B12 on diabetic neuropathy: systematic review of clinical controlled trials. Acta Neurol Taiwan 14: 48–54. PubMed: 16008162. 16008162

[11] Stracke H, Lindemann A, Federlin K (1996) A benfotiamine-vitamin B combination in treatment of diabetic polyneuropathy. Exp Clin Endocrinol Diabetes 104: 311–316. PubMed: 8886748. 10.1055/s-0029-1211460 8886748

[12] Yaqub BA, Siddique A, Sulimani R (1992) Effects of methylcobalamin on diabetic neuropathy. Clin Neurol Neurosurg 94: 105–111. PubMed: 1324807. 10.1016/0303-8467(92)90066-c 1324807

[13] Sigal RJ, Kenny GP, Boulé NG, Wells GA, Prud'homme D, et al.. (2007) Effects of aerobic training, resistance training, or both on glycemic control in type 2 diabetes: a randomized trial. Ann Intern Med 147: 357–369. PubMed: 17876019. 10.7326/0003-4819-147-6-200709180-00005 17876019

[14] Kluding PM, Pasnoor M, Singh R, Jernigan S, Farmer K, et al (2012) The effect of exercise on neuropathic symptoms, nerve function, and cutaneous innervation in people with diabetic peripheral neuropathy. J Diabetes Complications26: 424–429 10.1016/j.jdiacomp.2012.05.007.PubMed:22717465 PMC3436981 22717465

[15] Balducci S, Iacobellis G, Parisi L, Di Biase N, Calandriello E, et al.. (2006) Exercise training can modify the natural history of diabetic peripheral neuropathy. J Diabetes Complications 20: 216–23. PubMed: 16798472. 10.1016/j.jdiacomp.2005.07.005 16798472

[16] WU QL, Liang XC, J N, Luan S, Cui LY, et al (2012) Clinical Efficacy Observation of Diabetic Peripheral Neuropathy Treated with Jinmaitong Capsules. World Journal of Integrated Traditional and Western Medicine 7: 860–865.

[17] Zhang Q (2011) Intervention of Jinmaitong to Oxidative Stress in Dorsal Root Ganglion of Diabetic Rats. MPhil thesis, Beijing University of Chinese Medicine.

[18] Ji JL, Chen DS (2009) The Experiment Study of Nourishing Yin, Invigorating Qi, Extinguishing Wind, Promoting Blood Circulation and Draining Collateral Method of the Sciatic Nerve Schwann Cells Apoptosis of DM Rats. Journal of Emergency in Traditional Chinese Medicine 8: 1304–1306.

[19] Zhang XK, Cui LF (2005) Experimental research of Xiaoketongbi particles in preventing and treating early diabetic peripheral neuropathy. Shanxi Journal of Traditional Chinese Medicine 26: 728–729.

[20] Gao B, Su XL, Bai SY (2005) An experimental study of the expression of protein kinase C in diabetic peripheral neuropathy and chinese medicinal herbs interposition. Medical Journal of Liaoning 19: 12–14.

[21] Chen ZQ, Li JY, Huang J (2010) Protective action and mechanism of Jiaweibugan decoction on diabetic peripheral neuropathy rats. Shandong Medical Journal 50: 9–11.

[22] Ma ST (2005) The mechanism of Xiaoketongbi particles in protective effect of experimental diabetic neuropathy. Sichuan Journal of Physiological Sciences 27: 103–105.

[23] Wu J (2010) Experimental investigation of the protective effect on the diabetic peripheral neuropathy of SJT. Chongqing Medicine 39: 1377–1379.

[24] Zeng JZ, Dong KL, Li GC (2005) Effect of xiaokeling concentration fluid on mRNA expression of insulin-like growth factor-1 in sciatic nerve of Streptozotocin-induced diabetic rats. Journal of Central South University (Medical Sciences) 30: 49. 15871187

[25] Xue HL, Wang WJ, Chen JQ (2005) Effects of Shenmai Huoxue Decoction on early diabetic peripheral neuropathy in rats. Journal of Chinese Integrative Medicine. 3: 31–34. 10.3736/jcim20050110 15644157

[26] Wang MX, Kuang YX (2010) Experimental Study on the Effect of Tongluo Tangtai Prescription on Serum TNF-á of Rats with DPN. Henan Traditional Chinese Medicine 30: 354–350.

[27] Sun W, Miao B, Wang XC, Duan JH, Ye X, et al (2012) Gastrodin inhibits allodynia and hyperalgesia in painful diabetic neuropathy rats by decreasing excitability of nociceptive primary sensory neurons. PLoS One 7: e39647 10.1371/journal.pone.0039647.PubMed:22761855 22761855 PMC3382466

[28] Xu HB, Jiang RH, Chen XZ, Li L (2012) Chinese herbal medicine in treatment of diabetic peripheral neuropathy: a systematic review and meta-analysis. J Ethnopharmacol 143: 701–708 10.1016/j.jep.2012.07.034.PubMed:22885132 22885132

[29] Chen W, Zhang Y, Liu JP (2011) Chinese herbal medicine for diabetic peripheral neuropathy. Cochrane Database Syst Rev. 2011 (6): CD007796 10.1002/14651858.CD007796.PubMed:21678369 21678369

[30] Chen W, Luo YF, Liu JP (2011) Topical herbal medicine for treatment of diabetic peripheral neuropathy: a systematic review of randomized controlled trials. Forsch Komplementmed 18: 134–145 10.1159/000328457.PubMed:21701182 21701182

[31] World Health Organization (1999) Definition, Diagnosis and Classification of Diabetes Mellitus and its Complications: Report of a WHO Consultation. Part 1: Diagnosis and Classification of Diabetes Mellitus. Geneva: World Health Org. 3 p.

[32] American Diabetes Association (1997) clinical practice recommendations 1997. Diabetes Care Suppl 1: S1–70. PubMed: 9028710. 9028710

[33] Zheng XY (2002) Guideline for Clinical Trials of New Patent Chinese Medicines. 1st edition. Beijing: China medical science press. 233–237 p.

[34] Jadad AR, Moore RA, Carroll D, Jenkinson C, Reynolds DJ, et al.. (1996) Assessing the quality of reports of randomized clinical trials: is blinding necessary? Control Clin Trials 17: 1–12. PubMed: 8721797. 10.1016/0197-2456(95)00134-4 8721797

[35] Furlan AD, Pennick V, Bombardier C, van Tulder M; Editorial Board (2009) Cochrane Back Review Group (2009) 2009 Updated method guidelines for systematic reviews in the cochrane back review group. Spine 34: 1929–1941 10.1097/BRS.0b013e3181b1c99f.PubMed:19680101 19680101

[36] Hu JP (2012) The clinical observation of GuiZhiShaoYaoZhiMu Decoction in the treatment of Diabetic Peripheral Neuropathy. MPhil thesis, Guangzhou University of Chinese Medicine.

[37] Chen L (2011) The clinical observation of Xiaoketongluo Capsule in the treatment of Diabetic Peripheral Neuropathy. MPhil thesis, Beijing University of Chinese Medicine.

[38] Cao Y (2011) The Clinical study on treatment of diabetic peripheral neuropathy with Yiqiwenyanghuoxue Decoction. MPhil thesis, Shandong University of Traditional Chinese Medicine.

[39] Li L (2011) The clinical study about the research of efficacy for treating diabetic peripheral neuropathy (DPN) with tonifying qi huoxue away bi-complex Soup. MPhil thesis, Nanjing University of Chinese Medicine.

[40] Zhou XH (2010) The clinical study on the effect of boosting qi and nourishing yin, quickening the blood and transforming stasis to SNCV of patients with DPN. MPhil thesis, Chengdu University of Traditional Chinese Medicine.

[41] Zhang TJ (2008) The effect and mechanism of Tangluoning Decoction in the treatment of diabetic peripheral neuropathy. PhD thesis, Beijing University of Chinese Medicine.

[42] Liu SG (2004) Clinical study on the treatment of diabetic peripheral neuropathy with Tangluotong. MPhil thesis, Fujian College of Traditional Chinese Medicine.

[43] Heng XP (2004) Clinical study on Tangluotong for treatment of 60 cases of diabetic peripheral neuropathy. Journal of Traditional Chinese Medicine 45: 917–920.

[44] Li YZ (2005) The effect and mechanism of Tang ping luo tong yin on diabetes peripheral neuropathy. MPhil thesis, Hebei Medical University.

[45] Liu Q (2005) The clinical effect of Jiawei Huangqiguizhiwutang on the diabetic peripheral neuropathy. MPhil thesis, Chengdu University of Traditional Chinese Medicine.

[46] De Angelis C, Drazen JM, Frizelle FA, Haug C, Hoey J, et al.. (2004) International Committee of Medical Journal Editors. Clinical trial registration: a statement from the International Committee of Medical Journal. New England Journal of Medicine 351: 1250–1251. PubMed: 15356289. 10.1056/NEJMe048225 15356289

[47] Gagnier JJ, Boon H, Rochon P, Moher D, Barnes J, et al.; CONSORT Group. Reporting randomized, controlled trials of herbal interventions: an elaborated CONSORT statement. Ann Intern Med 2006, 144: 364–367. PubMed: 16520478. 10.7326/0003-4819-144-5-200603070-00013 16520478

[48] Kjaergard LL, Villumsen J, Gluud C (2001) Reported methodologic quality and discrepancies between large and small randomized trials in meta-analyses. Ann Intern Med 135: 982–989. PubMed: 11730399. 10.7326/0003-4819-135-11-200112040-00010 11730399

[49] Wang Y, Xie CL, Fu DL, Lu L, Lin Y, et al (2011) Clinical efficacy and safety of Chinese herbal medicine for Wilson's disease: a systematic review of 9 randomized controlled trials. Complement Ther Med 20: 143–154 10.1016/j.ctim.2011.12.004.PubMed:22500664 22500664

[50] Schulz KF, Altman DG, Moher D (2010) CONSORT Group (2010) CONSORT 2010 statement: updated guidelines for reporting parallel group randomized trials. Ann Intern Med 152: 726–732 10.7326/0003-4819-152-11-201006010-00232.PubMed:20335313 20335313

[51] Moher D, Hopewell S, Schulz KF, Montori V, Gotzsche PC, et al (2010) CONSORT 2010 explanation and elaboration: updated guidelines for reporting parallel group randomized trial. BMJ 340: c869 10.1136/bmj.c869.PubMed:20332511 20332511 PMC2844943

