# Supplementary material for: Chinese Herbal Medicine for Diabetic Peripheral Neuropathy: An Updated Meta-Analysis of 10 High-Quality Randomized Controlled Studies
Source: PLoS One. 2013 Oct 16;8(10):e76113. doi: 10.1371/journal.pone.0076113 (PMC3797714; doi:10.1371/journal.pone.0076113)
Supplement: Appendix S1 — Search strategies. (DOCX) [file pone.0076113.s001.docx]

**Appendix 1. Search strategy for Pubmed**

1. exp diabetes mellitus/

2. diabet$.mp.

3. 1 or 2

4. neuropath$.mp.

5. polyneuropath$.mp

6. exp peripheral nervous system diseases/

7. or/4-6

8. 3 and 7

9. exp diabetic neuropathies/

10. diabetic neuropath$.mp.

11. diabetic polyneuropath$.mp.

12. or/9-11

13. 8 or 12

14. chinese herbal/ or plants medicinal/ or medicine, herbal/

15. Medicine, Chinese Traditional/

16. medicine, oriental tradition/

17. (chinese adj7 (traditional or medicine$)).tw.

18. (herb or herbs or herbal).mp.

19. (plant or plants).mp.

20. (traditional adj8 medicine$).tw.

21. or/14-20

22. (Randomized or randomized or randomly or random order or random sequence or random allocation or randomly allocated or at random or controlled clinical trial$).mp. [mp =title, original title, abstract, name of substance word, subject heading word]

23. Clinical trial.pt.

24. 22 or 23

25. exp animals/ not humans.sh.

26. 24 not 25

27. 13 and 21 and 26
